# Prevalence of post-traumatic stress disorder risk post-COVID-19 in 12 countries in Latin America: a cross-sectional survey

**DOI:** 10.3389/fpubh.2023.1302694

**Published:** 2024-01-08

**Authors:** Christian R. Mejia, Víctor Serna-Alarcón, Martín A. Vilela-Estrada, Jose Armada, Milward Ubillus, Jose Beraún-Barrantes, Aldo Álvarez-Risco, Shyla Del-Aguila-Arcentales, Neal M. Davies, Jaime A. Yáñez

**Affiliations:** ^1^Universidad de Huánuco, Huánuco, Peru; ^2^Universidad Privada Antenor Orrego, Piura, Peru; ^3^Hospital Regional José Cayetano Heredia, EsSalud, Piura, Peru; ^4^Universidad Continental, Lima, Peru; ^5^Universidad Tecnológica del Perú, Lima, Peru; ^6^Escuela de Posgrado, Universidad San Ignacio de Loyola, Lima, Peru; ^7^Faculty of Pharmacy and Pharmaceutical Sciences, University of Alberta, Edmonton, AB, Canada; ^8^Asociación Médica de Investigación y Servicios en Salud, Lima, Peru; ^9^Universidad Peruana de Ciencias Aplicadas, Facultad de Educación, Carrera de Educación y Gestión del Aprendizaje, Lima, Peru

**Keywords:** COVID-19, Latin America, mental health, Peru, post-traumatic stress disorder, PTSD

## Abstract

**Introduction:**

Latin America was the region most affected by COVID-19 in the second quarter of 2020, and consequently, the impact on mental health requires evaluation. The aim of this study was to assess the risk of post-traumatic stress disorder (PTSD) caused by bereavement due to COVID-19 in 12 countries in Latin America.

**Methods:**

The current study was an analytical cross-sectional study. Validated tests were applied for PTSD, depression, anxiety, and stress (DASS-21), questions about the respondent’s condition or their environment, and demographic questions, as well as the length of the mourning period of suffering.

**Results:**

The outcomes demonstrated that the PTSD risk increased for women (*p* < 0.001), when a friend or acquaintance had COVID-19 (*p* = 0.002), when a close relative died from COVID-19 (*p* = 0.010), having severe depression (*p* <0.001), severe anxiety (*p*  <0.001), severe stress (*p*  <0.001), residing in Chile (*p*  <0.001), Paraguay (*p*  <0.001), Bolivia (*p*  <0.001), Costa Rica (*p*  <0.001) or El Salvador (*p*  = 0.005). On the other hand, there was less risk of PTSD at an older age (*p*  <0.001) or if respondents had a sentimental partner (*p*  = 0.025). In the case of severe PTSD, there was a greater gender risk for women (*p*  <0.001), a close relative dying from COVID-19 (*p*  = 0.017), having severe depression (*p*  <0.001), severe anxiety (*p*  <0.001), severe stress (*p*  <0.001), residing in Chile (*p*  <0.001), Paraguay (*p*  <0.001), Bolivia (*p*  <0.001) and Costa Rica (*p*  = 0.002). It was also observed that there was less risk of severe PTSD at an older age demographic (*p*  <0.001).

**Discussion:**

It can be concluded that the percentages of PTSD are high in its clinical presentation as severe, especially among Latin American women.

## Introduction

COVID-19 is an infectious illness caused by the SARS-CoV-2 virus. Most infected people with the virus present mild to moderate respiratory symptoms and recover without treatment requiring hospitalization. However, many people can also become severely ill and require medical attention (1). COVID-19 has been a major global socioeconomic and health problem comparable to what could be caused by a large-scale war (2). The beginning of this pandemic arose at the end of 2019 (3, 4), having a significant effect on Europe during the first quarter of 2020 (5) and affecting the North American continent in the subsequent months (6–8). The pandemic caused significant morbidity and mortality in millions of patients, as well as social and economic repercussions such as isolation, restrictions, and a host of additional problems and sequelae (9, 10). In addition, through mainstream and social media, additional fear and panic were generated and exacerbated in the populations (11–13).

There is evidence that several illnesses, such as hypertension (14), diabetes (15, 16), Ebola (17–19), SARS (20), dengue (21), H7N9 (22), and H1N1 (23), can generate post-traumatic stress disorder (PTSD). PTSD has been defined as a mental health condition that can affect people who have experienced or witnessed a traumatic event, series of events, or set of circumstances. Such an event can be emotionally or physically harmful or life-threatening, which can lead to symptoms that affect the mental, physical, social, and/or spiritual well-being of an individual (24).

The measurement of PTSD using DASS-21 (25–28) has previously been undertaken. However, there have been few studies linking PTSD and COVID-19 with survivors (29, 30) and healthcare workers (31, 32).

The possible effect of the COVID-19 pandemic on population mental health (33), especially for those who suffered from the disease and those who had relatives who were infected or even died, has been addressed (34). The COVID-19 pandemic was characterized by some relevant features that increase the risk of PTSD, such as an often-unpredictable course of the disease, high mortality rates (35–37), lack of knowledge and preventive practices (38), and lack of effective treatment, treatment guidelines, and the appearance of viral variants (39). PTSD related to COVID-19 has been reported in healthcare workers (31, 32) and the general public (40, 41), including perinatal women (30).

Recent studies have highlighted the significant occurrence of PTSD among individuals affected by COVID-19, with health professionals on the front lines facing heightened risks due to intense and prolonged exposure to trauma. The nature of one’s vocation, such as the specific health profession and the unit of work within the healthcare system, plays a crucial role in determining susceptibility to PTSD. The prevalence of PTSD among healthcare workers during the COVID-19 pandemic has been reported to be 13.52% globally (42). A study comparing Italian healthcare professionals to a control group of the general population found that the prevalence of self-reported PTSD symptoms caused by the COVID-19 pandemic was high (43). Another study reported that the prevalence of PTSD among intensive care unit (ICU) professionals increased to 73.3% following the COVID-19 health crisis (44). A review of the literature highlighted the high prevalence of PTSD, especially among healthcare professionals who work in COVID-19 wards (45). A multi-centered cross-sectional study in Northwest Ethiopia found the prevalence of PTSD among healthcare providers during the COVID-19 pandemic to be 55.1% (46).

These findings highlight the significant effect of the COVID-19 pandemic on the mental health of healthcare professionals, emphasizing the need for standardized and culturally sensitive measures to assess the true extent of PTSD in different populations. The nature of one’s work and geographical location have been identified as key factors influencing susceptibility to PTSD. Additionally, age emerges as a key moderator, with younger individuals often exhibiting different vulnerability patterns compared to their older counterparts. The geographical aspect is also crucial, as PTSD prevalence can vary across continents, reflecting diverse cultural and contextual factors. The choice of assessment tools can influence reported prevalence rates, emphasizing the importance of standardized and culturally sensitive measures to capture the true extent of PTSD in different populations. PTSD varies widely, with a lifetime prevalence ranging from 6.1 to 9.2% in national samples (42, 47, 48). In the United States, the 1 year prevalence of PTSD was estimated at 6.7% among male veterans and 11.7% among female veterans, and the lifetime prevalence was reported to range from 3.4 to 26.9% among civilians.

Factors, such as female sex, lower income, younger age, and behavioral health conditions, were identified as risk factors for PTSD (47, 48). These findings highlight the importance of understanding significant moderators of PTSD prevalence for tailoring interventions and support strategies for those at risk of or experiencing PTSD.

In highly affected populations, such as Peru, which became the country with the world’s highest per-capita COVID-19-related mortality (49, 50), significant mental health issues have arisen. In addition, other Latin American countries have also been significantly affected (51–53). However, to the best of our knowledge, there has not been any published study that addressed early PTSD in a large Latin American population that could serve as a baseline of PTSD levels at the different stages of the pandemic: surge of infodemic, early quarantine, various waves, and prior to the massive distribution of vaccines (54, 55).

The objective of this study was to evaluate the risk of PTSD according to the immediate environment’s suffering or grief after exposure to COVID-19 in 12 countries in Latin America.

## Materials and methods

### Study design

We conducted an online cross-sectional multicenter survey in Spanish-speaking countries (Peru, Chile, Paraguay, Mexico, Colombia, Bolivia, Panama, Ecuador, Costa Rica, El Salvador, Honduras, and Guatemala) between June 7, 2021 and August 30, 2021, which were the pandemic months with the most significant effect in Latin America. The design was based on the fact that each respondent was approached only once and that descriptive and analytical results were generated. It was determined that a minimum sample size of 3,204 was necessary to achieve a minimum percentage difference of 2.5% (49.0% vs. 51.5%), a statistical power of 80%, and a confidence level of 95% (data not shown). The sample size was calculated using power analysis (56) and based on a previous study in Peru that assessed PTSD after a natural phenomenon (57). Residents of the countries in question were included who reported that they remained in those territories during the time of the pandemic and who agreed to participate in the research. Duplicate responses were excluded, as were those who did not answer all the questions on the measured scales, those who did not have complete personal information, or those who presented anomalous response patterns (more than 4,000 surveys were excluded for all these reasons). The survey consisted of an online questionnaire in Google Surveys that was sent via WhatsApp, Messenger, and Facebook, and it was configured to enable the submission of an email at the end of the survey so that the investigation group could ensure that individual data were submitted. The survey was generated only online because, at the time of sampling, there were still individual restrictions on circulating freely in public places. In addition, online sampling was selected to prevent the surveyors from becoming infected and spreading the disease even further. The shared questionnaire was made anonymous, ensuring data confidentiality and reliability; each participant was informed in the first part of the questionnaire that they were free to participate, that they were free to withdraw if they liked, and that, by not asking for identifying data, we would not be able to know or divulge their identity. This survey was undertaken in the Spanish language since we surveyed only Spanish-speaking countries in Latin America in laboratory-confirmed cases. The survey was performed from June 7, 2021 to August 30, 2021, after approximately 3–5 months of lockdown and social distancing measures in Latin America due to the COVID-19 outbreak. At the beginning of the survey (June 7, 2021), the number of confirmed COVID-19 cases in the surveyed countries totaled 625,495 and the number of confirmed deaths was 36,287, while, at the end of the survey (August 30, 2021), the number of confirmed cases increased to 2,685,447 and the deaths increased to 136,068. We surveyed the public with adults (over 18 years old) in all countries that participated in the survey (Peru, Chile, Paraguay, Mexico, Colombia, Bolivia, Panama, Ecuador, Costa Rica, El Salvador, Honduras, and Guatemala). Participants were recruited through the FELSOCEM-ASOMEDISS COVID-19 Latam (which is an organization of physicians and medical students from almost every country in Latin America), a network of investigators that includes physicians, health professionals, and students performing COVID-19 social epidemiological studies in Peru and Latin America. Each collaborator verified that the contact to whom they sent the virtual survey could answer it adequately and that they were willing to resolve the doubts of the participants.

### Outcomes and covariates

The survey (Annex 1) included 46 questions, of which 13 were demographic, 21 were from the DASS-21 test, and 12 were related to suffering from post-traumatic stress and also having this pathology, but in a severe stage (with suicidal ideation), both by applying the Short Post-traumatic Stress Disorder Rating Interview (SPRINT-E) created in 2001 to measure the symptoms of this pathology (58). The instrument has been used in Chile, where it obtained a value of 0.92 for Cronbach’s alpha (59); in the current study, a Cronbach’s alpha of 0.93 was obtained. For anxiety, stress, and depression, the DASS-21 test was used, which has been validated and used in multiple settings (60), where the severe category was used for each case and a Cronbach’s alpha of 0.96 was obtained.

There were multiple exposure variables as follows: (a) if a friend or close acquaintance was afflicted with COVID-19; (b) if a friend or acquaintance died from COVID-19; (c) if someone at home was sick from COVID-19; (d) if a family member was not home, they became ill from COVID-19; (e) if a close relative died from COVID-19; (f) if a distant relative died from COVID-19; and (g) if it was suspected or it was very likely that someone had COVID-19 (according to the report of having symptoms, but not a confirmatory test) and had or became ill with COVID-19 (confirmed with rapid or molecular test). The demographic questions included the gender (male or female), age (in completed years), if they had a romantic partner (yes or no), job status, type of job, level of education (university / postgraduate or a lower academic level), and the country of residence (of the 12 countries already mentioned). There were multiple exposure variables as follows: if a friend or close acquaintance became sick from COVID-19, if a friend or acquaintance died from COVID-19, if someone at home fell ill from COVID-19, if a family member who was not at home became ill from COVID-19, if a close relative died from COVID-19, if a distant relative died from COVID-19 if it was suspected or it was very likely that they had contracted COVID-19 (according to the report of having symptoms, but not a confirmatory test) and had or became ill with COVID-19 (confirmed with rapid or molecular test).

### Ethics approval

The research has the approval of the Universidad Privada Antenor Orrego (UPAO) Bioethics Committee, a human ethics committee in Peru (Resolution of the Bioethics Committee No. 0240-2020-UPAO). The same endorsement could not be made in other countries since the pandemic generated the closure of most institutions that housed researchers during the pandemic. After obtaining approval in Peru, we proceeded with the respondents in the various countries; in each one, a non-random sample was obtained (due to the difficulty of having official lists). The participants remained anonymous and could finish the survey at any time, and their information was kept confidential. All the survey participants were well-versed in the study intentions and were required to consent before enrollment. The participants were not involved in any of the planning, execution, or reporting stages of the study.

### Statistical analysis

Data analysis was done in STATA version 14 (Stata Corp.) with a significance level of *p* < 0.05. The instrument validity was assessed with the known-groups validity approach by fitting multivariate analysis. Univariate statistics were represented with frequencies and percentages for categorical variables. A description of the variables was made in each country, showing the percentages of the dependent variables and anxiety, depression, and stress (in their severe form). Then, the bivariate models were carried out, where each independent variable was crossed with the two dependent variables, from which they were statistically significant (*p* < 0.05), and they were entered into the multivariate model. For analytical statistics, adjusted prevalence ratios (aPR) and 95% confidence intervals (CIs) were obtained using generalized linear models (GLM), with the Poisson family, log-link function, and models for robust variances to adjust for the large sample size..

## Results

### Sociodemographic characteristics of the respondents

The survey was sent to 9,000 people in Peru, Chile, Paraguay, Mexico, Colombia, Bolivia, Panama, Ecuador, Costa Rica, El Salvador, Honduras, and Guatemala to achieve the minimum sample size of 3,204 calculated based on power analysis. Out of the 9,000 surveys sent online, we received 8,194 responses indicating a 91.0% response rate. Most participants were women (4,854 [59.2%]), were those aged 18–89 years, were single (6,699 [81.8%]), had some university studies or higher (5,750 [70.2%]), and had a romantic partner (3,669 [44.8%]). The country evaluated with the highest response was Peru, with 4,026 surveys, and it had the most deaths/infections (120). The population was also evaluated in Chile (738), Mexico (647), Paraguay (583), Colombia (435), Bolivia (385), Panama (374), Ecuador (279), Costa Rica (256), El Salvador (199), Honduras (162), and Guatemala (110).

**Figure 1:**
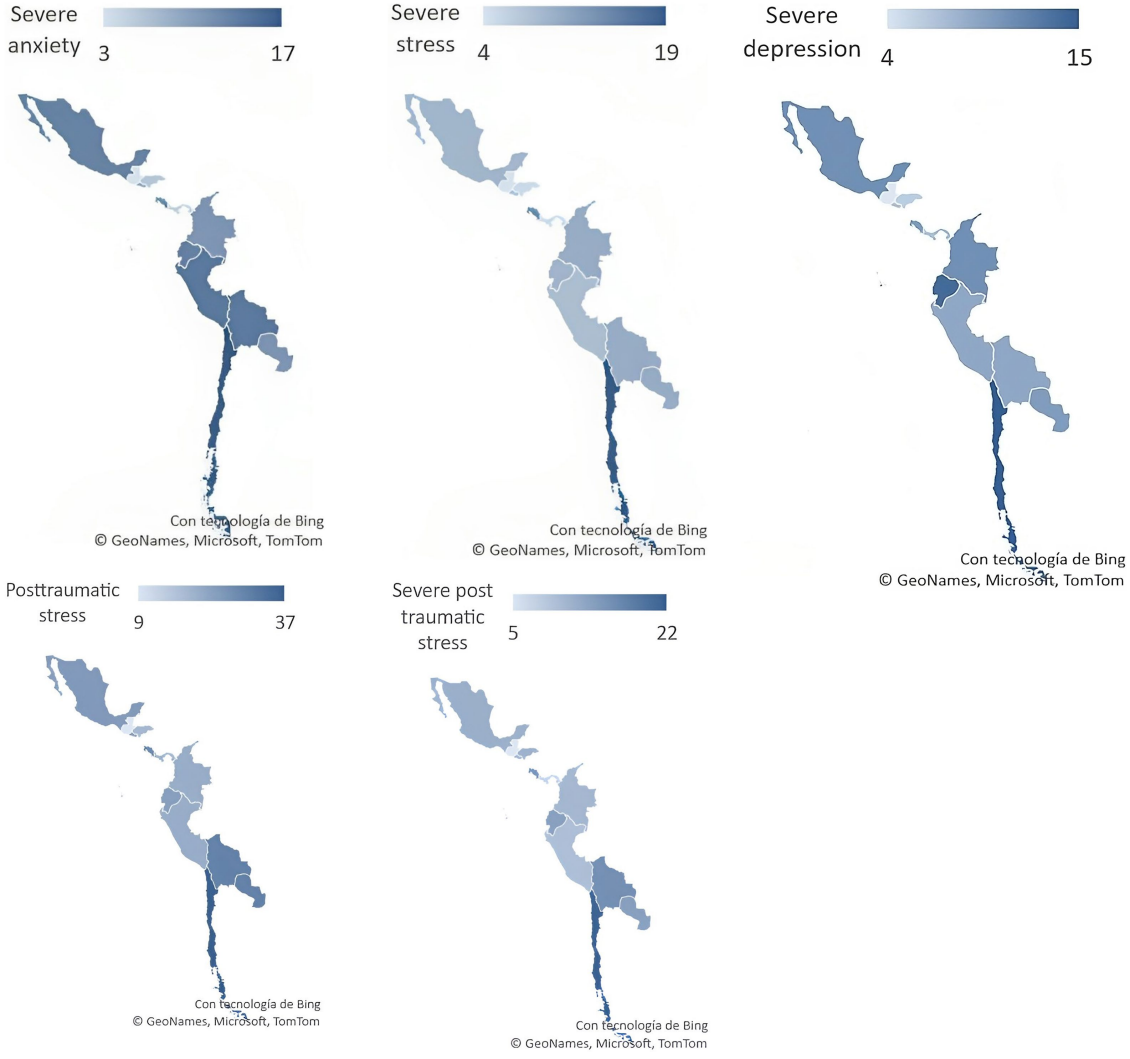
Severe anxiety, severe stress, severe depression, PTSD, and severe PTSD in 12 Latin American countries during the COVID-19 pandemic.

Of the 8,194 respondents in Latin America, there was a higher frequency of severe episodes of anxiety (Chile 17%, Peru 14%, and Bolivia 14%), stress (Chile 19% and Costa Rica 14%), and depression (Chile 15% and Ecuador 14%). In addition, there was a high frequency of PTSD (Chile 37%, Paraguay 30%, and Bolivia 30%) and severe PTSD (Chile 22%, Costa Rica 16%, and Bolivia 16%; Figure 1).

In the bivariate analysis, it was determined that more PTSD or severe PTSD was observed among women (*p* < 0.001 for both), among those who had a friend or acquaintance with the diagnosis of COVID-19 (*p* < 0.001 for both), among those who had a relative outside the home with COVID-19 (*p* < 0.035 for both), among those who had a close relative who died from COVID-19 (*p* < 0.002 for both), among those whose distant relative died from COVID-19 (*p* < 0.001 for PTSD), if the respondent suspected COVID-19 (*p* = 0.002 for both), and if the respondent confirmed that they had COVID-19 (*p* = 0.008 for severe PTSD). Additionally, respondents who confirmed their own COVID-19 diagnosis had severe depression (*p* < 0.001 for both), severe anxiety (*p* < 0.001 for both), or severe stress (*p* < 0.001 for both). Similarly, compared to Peru, there was greater prevalence among those who resided in Chile (*p* < 0.001 for both), Mexico (*p* = 0.019 for PTSD), Paraguay (*p* < 0.002 for both), Bolivia (*p* < 0.001 for both), Costa Rica (*p* < 0.008 for both), or Guatemala (*p* = 0.005 for PTSD). On the contrary, the risk was reduced with advanced age (*p* < 0.001 for both) and by having a romantic partner (*p* < 0.001 for EPT) (Table 1).

**Table 1:** Bivariate analysis of risk factors for post-traumatic stress disorder (PTSD) and severe PTSD in 12 Latin American countries during the COVID-19 pandemic (*n* = 8,194).

Variables	Post-traumatic stress disorder (PTSD)	Severe PTSD
Women	1.80 (1.65–1.96) <0.001	1.94 (1.70–2.21) <0.001
Age (years)	0.97 (0.97–0.98) <0.001	0.97 (0.96–0.97) <0.001
University studies	1.01 (0.93–1.10) 0.836	1.09 (0.96–1.24) 0.175
Has a sentimental partner	0.86 (0.80–0.93) <0.001	0.91 (0.81–1.03) 0.126
Friend or acquaintance with COVID-19	1.26 (1.16–1.37) <0.001	1.31 (1.16–1.48) <0.001
Friend or acquaintance who died from COVID-19	1.07 (0.98–1.18) 0.132	1.07 (0.93–1.23) 0.321
Family at home with COVID-19	1.11 (0.93–1.33) 0.241	1.19 (0.92–1.54) 0.178
Family away from home with COVID-19	1.17 (1.07–1.28) 0.001	1.16 (1.01–1.34) 0.034
Close family died from COVID-19	1.32 (1.15–1.51) <0.001	1.41 (1.15–1.73) 0.001
Distant family died of COVID-19	1.23 (1.10–1.37) <0.001	1.03 (0.86–1.24) 0.720
Respondent could have COVID-19	1.25 (1.08–1.45) 0.002	1.39 (1.13–1.72) 0.002
Surveyed with confirmed COVID-19	1.19 (0.93–1.52) 0.159	1.55 (1.12–2.14) 0.008
With severe depression (DASS-21 test)	4.01 (3.77–4.27) <0.001	7.74 (7.00–8.56) <0.001
With severe anxiety (DASS-21 test)	3.85 (3.61–4.11) <0.001	6.48 (5.83–7.20) <0.001
With severe stress (DASS-21 test)	4.20 (3.95–4.46) <0.001	7.54 (6.82–8.33) <0.001
**Country of residence**		
Peru	Comparison category	Comparison category
Chile	1.72 (1.54–1.93) <0.001	2.14 (1.82–2.52) <0.001
Mexico	1.19 (1.03–1.37) 0.019	1.13 (0.90–1.42) 0.293
Paraguay	1.38 (1.20–1.59) <0.001	1.44 (1.16–1.78) 0.001
Colombia	0.99 (0.82–1.20) 0.938	1.04 (0.78–1.38) 0.784
Bolivia	1.39 (1.18–1.64) <0.001	1.55 (1.21–1.98) <0.001
Panama	0.97 (0.78–1.19) 0.745	0.80 (0.56–1.13) 0.206
Ecuador	1.11 (0.89–1.38) 0.350	1.31 (0.96–1.78) 0.085
Costa Rica	1.36 (1.11–1.66) 0.003	1.50 (1.12–2.03) 0.007
El Salvador	1.15 (0.90–1.48) 0.258	1.11 (0.75–1.65) 0.595
Honduras	0.87 (0.63–1.21) 0.402	1.13 (0.73–1.74) 0.580
Guatemala	0.43 (0.24–0.77) 0.005	0.53 (0.24–1.15) 0.107

Relative risks (left), 95% confidence intervals (within parentheses), and *p*-values (right) were obtained with generalized linear models, the log-link function, and models for robust variances.

Multivariate analysis was performed, and a higher risk of PTSD was found among women (*p* < 0.001), among those who had a friend or acquaintance with a diagnosis of COVID-19 (*p* = 0.002), among those whose close relative died from COVID-19 (*p* = 0.010), among those who had severe depression (*p* < 0.001), severe anxiety (*p* < 0.001), or severe stress (*p* < 0.001) at the time of the survey, and among those who resided in Chile (*p* < 0.001), Paraguay (*p* < 0.001), Bolivia (*p* < 0.001), Costa Rica (*p* < 0.001), or El Salvador (*p* = 0.005). On the other hand, there was lower risk of PTSD at an advanced age (*p* < 0.001) or if the respondent had a sentimental partner (*p* = 0.025), adjusted for three variables (Table 2).

**Table 2:** Multivariate analysis of risk factors for PTSD and severe PTSD in 12 Latin-American countries during the COVID-19 pandemic (*n* = 8,194).

Variables	Post-traumatic stress disorder (PTSD)	Severe PTSD
Women	1.55 (1.42–1.68) <0.001	1.51 (1.34–1.71) <0.001
Age (years)	0.98 (0.98–0.98) <0.001	0.98 (0.98–0.99) <0.001
University studies	Did not enter the model	Did not enter the model
Has a sentimental partner	0.92 (0.85–0.99) 0.025	Did not enter the model
Friend or acquaintance with COVID-19	1.14 (1.05–1.23) 0.002	1.12 (1.00–1.27) 0.059
Friend or acquaintance who died from COVID-19	Did not enter the model	Did not enter the model
Family at home with COVID-19	Did not enter the model	Did not enter the model
Family away from home with COVID-19	1.03 (0.94–1.13) 0.483	0.99 (0.86–1.13) 0.832
Close family died from COVID-19	1.20 (1.04–1.38) 0.010	1.28 (1.05–1.58) 0.017
Distant family died of COVID-19	1.06 (0.95–1.19) 0.299	Did not enter the model
Respondent could have COVID-19	1.01 (0.88–1.16) 0.860	1.05 (0.87–1.27) 0.631
Surveyed with confirmed COVID-19	Did not enter the model	1.13 (0.81–1.57) 0.479
With severe depression (DASS-21 test)	1.74 (1.57–1.94) <0.001	2.82 (2.33–3.40) <0.001
With severe anxiety (DASS-21 test)	1.89 (1.68–2.11) <0.001	1.99 (1.63–2.42) <0.001
With severe stress (DASS-21 test)	1.50 (1.34–1.68) <0.001	1.87 (1.54–2.26) <0.001
**Country of residence**		
Peru	Comparison category	Comparison category
Chile	1.54 (1.39–1.70) <0.001	1.67 (1.44–1.93) <0.001
Mexico	1.07 (0.94–1.22) 0.303	1.00 (0.81–1.24) 0.968
Paraguay	1.45 (1.27–1.66) <0.001	1.48 (1.21–1.80) <0.001
Colombia	1.00 (0.85–1.19) 0.956	1.04 (0.82–1.32) 0.731
Bolivia	1.37 (1.18–1.59) <0.001	1.56 (1.25–1.93) <0.001
Panamá	1.17 (0.96–1.41) 0.112	1.06 (0.77–1.45) 0.727
Ecuador	1.01 (0.83–1.22) 0.937	1.11 (0.86–1.43) 0.410
Costa Rica	1.46 (1.20–1.77) <0.001	1.57 (1.18–2.08) 0.002
El Salvador	1.40 (1.11–1.77) 0.005	1.39 (0.97–2.00) 0.073
Honduras	0.96 (0.71–1.29) 0.763	1.38 (0.93–2.04) 0.111
Guatemala	0.60 (0.35–1.03) 0.066	0.84 (0.43–1.63) 0.605

Relative risks (left), 95% confidence intervals (within parentheses), and *p*-values (right) were obtained with generalized linear models, the log-link function, and models for robust variances.

For severe PTSD, there was a higher risk among women (*p* < 0.001), among those who had a close relative who died from COVID-19 (*p* = 0.017), among those who had severe depression (*p* < 0.001), severe anxiety (*p* < 0.001), and severe stress (*p* < 0.001), among those who resided in Chile (*p* < 0.001), Paraguay (*p* < 0.001), Bolivia (*p* < 0.001), and Costa Rica (*p* = 0.002); on the other hand, there was a lower risk of severe PTSD at an older age (*p* < 0.001), adjusted by four variables (Table 2).

## Discussion

Frequencies of PTSD and severe PTSD were found in up to one in three and one in five respondents, respectively. Despite not being able to extrapolate the results to the rest of the continent, these results are alarming due to the high number of people with an alteration in the mental health plane, without counting the large percentage of respondents who have thought about committing suicide. The findings of PTSD are approximately equivalent to those previously reported in other geographical locations; this is consistent with a meta-analysis of more than 60 studies published in different circumstances in countries of Europe, Asia, and North America, where a clinically significant prevalence of PTSD of 32% was recorded (61). Furthermore, the findings related to severe PTSD are striking since, even in China, the epicenter of the pandemic, and Italy, one of the countries initially most affected by COVID-19 (62), a much lower prevalence of PTSD was reported, and there have been very few cases of severe PTSD (40, 63). The timing of the measurement, access to information about the severity of the virus, and the educational level affected the results since it is evident that those with a lower level of education have a greater risk of psychological distress (64). All these variables should be further studied, as this is a fairly accurate approximation of the circumstances reflecting occurrences in the Latin-American population’s mental health arena.

Women were at increased risk of PTSD and severe PTSD, as observed in previous research (63, 65), which shows that women have been more susceptible to alterations caused by the coronavirus. In parts of Latin America, women are still associated with significant domestic work and caring for the home and its members, which also increased with the “lockdown” families had in their homes. In addition, women historically have occupied the roles of caregivers of the home, which, in some similar situations, translates into symptoms, such as insomnia, fatigue, anxiety, stress, and depression. Finally, it is often assumed that, in addition to their salaried jobs (66), women are subject to changes due to the crisis. Finally, cases have been reported of women who, due to the pandemic’s effects and measures, were forced to return to live with their domestic partner abusers, further increasing their access to support networks (67). This paragraph highlights the null gender perspective in the general measures taken during the pandemic, often overlooking their direct effects on the health of women.

At an older age, there was a lower risk of PTSD and severe PTSD. These results are striking since they are contrasted with other articles that present this age group as one of the highest risk groups. This difference can be explained by concerns about health complications for older adults and their close family members, along with comorbidities, the decrease in controls for chronic diseases during the pandemic, and the infantilization of their decisions in this same context (68). However, our study results can be further explained because older adults have already lived with these daily concerns and have been able to cope with a series of uncertain events, such as other epidemics (21). In addition to this, they tend to have limited access to social networks, which is considered a protective factor because they would avoid being affected by news that could cause anxiety, depression, and stress (69). The previous explanation does not mean that this is a group that must be in constant analysis to be able to detect the appearance of alarming symptoms, such as isolation, anxiety, excessive worries about the disease, excessive thinking about death, and other typical signs, which tend to go unnoticed due to the global contingency.

Those respondents who had a romantic partner were at lower risk of developing PTSD in the context of the COVID-19 pandemic. Our results are similar to those found in other studies applied to the general population of China (70, 71), Italy (72), Mexico (64), and Spain (73). The underlying factors that may explain this protective effect are real or perceived satisfaction, stability, understanding, attention, support, and emotional security. In turn, this effect depends on the quality of romantic relationships and communication (virtually or remotely in the first month and in person when the quarantines were lifted), the degree of alliance and commitment of the couple, mutual trust, and well-being generated (74). On the contrary, the marital status of widowed, separated, or divorced people has been reported as a risk factor for suffering from severe PTSD (75).

Severe stages of anxiety, depression, or stress have a higher risk of PTSD and severe PTSD, which can be rationalized as a determinant because each individual experiences and faces the same event differently (76). PTSD is a chronic mental illness that generally develops after being exposed to severe trauma, intrusive memories, distressing dreams, and a negative mood. It is estimated that approximately 6% of people exposed to psychological trauma go on to develop PTSD (57); according to this data and other references, it is considered that severe stages of anxiety, depression, or stress are risk factors for having PTSD and severe PTSD. These results are related to gender differences since it has been shown that women with some susceptibilities have a higher risk of being affected by PTSD (77).

Residing in different countries generated an increased risk of PTSD or severe PTSD compared to those who lived in Peru, which is the country with the most significant effect worldwide, showing that mental health could be impaired in multiple realities. These outcomes have been shown in studies of the area in different countries across several continents (78–84). Therefore, it is suggested that mental health measurement campaigns be generated in various settings as a baseline so that measures can be implemented that facilitate recovery, especially among the populations identified as being the most affected..

Some additional recommendations for future research and interventions to address the mental health concerns of people during pandemics or other high-stress situations include additional social and practical support. Literature reviews have concluded that social and practical support are important mechanisms for alleviating psychological distress and may be preferred to professional psychological support. This highlights the need for interventions that focus on providing social and practical support to individuals affected by pandemics (85). Furthermore, research on the effectiveness of mental health interventions during pandemics is growing. Future research should continue to evaluate the effectiveness of various interventions, including psychosocial interventions and the implementation of existing or new training programs, to address the mental health sequelae of pandemics (86). Moreover, addressing the psychological response of healthcare workers to medical pandemics is complicated. Future research should focus on developing interventions that specifically address the unique psychological challenges faced by healthcare workers during pandemics, including the effect of the pandemic on their personal, professional, and relational levels (87).

Additionally, when designing and implementing mental health interventions, it is important to consider cultural adaptations and the mental health workforce. Future research should explore the cultural and geographical constraints that may affect the effectiveness of mental health interventions during pandemics and the role of the mental health workforce in providing support to those most in need (86). Our recommendations highlight the need for future research and interventions that focus on providing social and practical support, evaluating the effectiveness of interventions, addressing the psychological response of healthcare workers, and considering cultural adaptations and the mental health workforce during pandemics.

The main implications of this research were, in general, the fact that we contributed to filling a gap that existed in terms of the immediate consequences in mental health to cause PTSD in a large population in Latin America as well as the main factors associated with it. The effects of the COVID-19 pandemic on other aspects of mental health in the medium and long term in Latin America have been reported (88–91).

All of these implications should serve the health sector, as well as the governing and academic institutions of each country. To evaluate the mental health of the population continuously, an investigation is recommended. Post-traumatic stress in various populations can cause medium- to long-term effects and can be linked with an increase in suicides (92, 93). Therefore, it highlights the importance of generating early detection, intervention, and treatment programs.

The study had an information bias because it was based on the respondents’ information, especially those who suffered (or had suspicions) if someone in their environment became ill or died due to COVID-19. It is possible that some cases did not occur, but this issue is not crucial because we intended to capture the thoughts and mental effects of respondents. One limitation was a small sample size in some countries, which requires a situational analysis to extrapolate the results. Our objective was to show the reality of a time period related to the significant mortality peaks in each country during the pandemic. This can be considered the main strength of the research because it evaluates the incidence of PTSD early on in the pandemic in a large population in multiple Latin American countries with different socioeconomic realities and various mental health coping mechanisms. Further research is warranted to determine the current incidence of PTSD and how it might be a further detriment to the overall mental health of the Latin American population.

## Conclusion

It can be concluded that, in this cross-sectional survey, the prevalence percentages of PTSD risk are high in its severe presentation post-COVID-19 in 12 countries in Latin America. This risk increased among women if they had a friend or acquaintance who suffered from COVID-19 or a close relative who died from COVID-19; it also increased among those who had severe anxiety, depression, or stress and resided in Latin American countries. On the contrary, older age or having a romantic partner appears to reduce the risk of PTSD, as determined in this cross-sectional survey.

## Data availability statement

The raw data supporting the conclusions of this article will be made available by the authors, without undue reservation.

## Ethics statement

The studies involving humans were approved by Universidad Privada Antenor Orrego (No. 0240-2020-UPAO). The studies were conducted in accordance with the local legislation and institutional requirements. The participants provided their written informed consent to participate in this study.

## Author contributions

CM: Conceptualization, Formal analysis, Investigation, Methodology, Software, Supervision, Visualization, Writing – original draft, Writing – review & editing. VS-A: Investigation, Methodology, Project administration, Validation, Visualization, Writing – original draft, Writing – review & editing. MV-E: Conceptualization, Investigation, Supervision, Visualization, Writing – original draft, Writing – review & editing. JA: Investigation, Methodology, Supervision, Visualization, Writing – original draft, Writing – review & editing. MU: Resources, Validation, Visualization, Writing – original draft, Writing – review & editing. JB-B: Resources, Supervision, Visualization, Writing – original draft, Writing – review & editing. AÁ-R: Formal analysis, Methodology, Visualization, Writing – original draft, Writing – review & editing. SD-A-A: Formal analysis, Visualization, Writing – original draft, Writing – review & editing. ND: Resources, Visualization, Writing – original draft, Writing – review & editing. JY: Supervision, Visualization, Writing – original draft, Writing – review & editing.
